# The Influence of Passive Ultrasonic Irrigation (PUI) on the Healing of Maxillary Sinusitis of Endodontic Origin (MSEO) After Non-Surgical Endodontic Treatment

**DOI:** 10.3390/jcm15062454

**Published:** 2026-03-23

**Authors:** Paweł Szczurowski, Michał Gontarz, Krzysztof Gronkiewicz, Piotr Majewski, Barbara Czopik

**Affiliations:** 1 Department of Cranio-Maxillofacial Surgery, Jagiellonian University Medical College, 30-688 Cracow, Poland; 2 Department of Dental Prosthetics and Orthodontics, Jagiellonian University Medical College, 31-155 Cracow, Poland; krzysztof.gronkiewicz@uj.edu.pl (K.G.);; 3 Private Endodontic Practice PS24, 30-654 Cracow, Poland

**Keywords:** maxillary sinusitis of endodontic origin, MSEO, endodontic treatment, maxillary sinusitis of dental origin, MSDO, PUI, passive ultrasonic irrigation, ultrasounds

## Abstract

**Background/Objectives**: Half of diagnosed unilateral maxillary sinusitis may have odontogenic origin, and root canal treatment (RCT) can be beneficial as a single-mode treatment for full resolution of maxillary sinusitis of endodontic origin (MSEO) symptoms. The aim of the study was to investigate the influence of passive ultrasonic irrigation (PUI) on the healing of MSEO after non-surgical endodontic treatment. **Methods**: A single-center, retrospective study was conducted on CBCT data and medical records of 240 patients, who underwent non-surgical endodontic treatment, performed by the same operator between 2016 and 2025. One hundred and thirty-six teeth entered the study due to inclusion/exclusion criteria. **Results**: Complete healing was observed in 75.74% of the cases (*n* = 103). The tooth most frequently associated with MSEO was the first upper molar (52.21%, *n* = 71). PUI was applied in 66.91% of the treatments (*n* = 91). PUI was an independent predictor of MSEO healing (*p* = 0.001; 95% CI [1.768; 9.136]). When PUI was introduced in RCT, complete healing of MSEO was observed in 84.62% of the cases (*n* = 77). PUI was independently associated with higher odds of radiological resolution of MSEO in univariate logistic regression (OR = 4.019) and multiple logistic regression (OR = 12.388) models. **Conclusions**: PUI application in the irrigation protocol was associated with higher rates of MSEO healing after non-surgical endodontic treatment (*p* = 0.001; 95% CI [1.768; 9.136]). The rate of MSEO healing after non-surgical RCT is high (75.74%); therefore it should be considered as a single-mode treatment option in cases of unilateral maxillary sinusitis.

## 1. Introduction

Recent studies report that up to 50% of unilateral maxillary sinusitis cases may have odontogenic origin [1]. Among those, most common dental etiologies causing maxillary sinusitis are chronic apical periodontitis (AP) [2], and when a periapical lesion existed, the prevalence of maxillary sinusitis of odontogenic origin (MSOO) reached 79% [3]. While studies vary, there is a slight female predominance in MSOO incidence, and this condition commonly affects patients in their 40s and 50s [4]. In order to highlight the significance of endodontic infection in the development of MSOO, the American Association of Endodontists (AAE) outlined maxillary sinusitis of endodontic origin (MSEO) in the category of dental-induced sinusitis [5]. The causal pathway linking intracanal disinfection to the resolution of proliferated sinus mucosa is rooted in elimination of the microbial source of MSEO [5]. The anatomical proximity of apical area of premolars and molars with maxillary sinus makes it possible for odontogenic pathogens and inflammatory mediators to spread from infected maxillary posterior teeth across the sinus floor, causing Schneiderian membrane hyperplasia [6]. With key risk factors, identified as failed RCT, the presence of AP, and the anatomical proximity of molar root apices to the maxillary sinus floor [4], MSEO should be in fact considered as an endodontic infection manifesting in the maxillary sinus, caused by the spread of bacteria from necrotic or infected maxillary posterior teeth [5]. Most of the studies consider MSEO, when a source of endodontic origin is adjacent to the sinus with sinusitis, forming two characteristic symptoms of the disease: PAM (periapical mucositis) and PAO (periapical osteoperiostitis) [5]. MSEO can manifest itself with both: dental and sinonasal symptoms, however, the clinical progression of the disease may not correlate with severity of the symptoms, as in most of the cases teeth involved in MSEO development are asymptomatic [2,5]. This emphasizes the role of radiological imaging, with Cone Beam Computed Tomography (CBCT) being a gold standard in MSEO diagnostics [5].

Few studies have examined the effects of root canal treatment (RCT) on maxillary sinusitis of endodontic origin, suggesting that it may be beneficial as a single-mode treatment for full resolution of the symptoms [7]. Moreover, we reported that rate of MSEO healing after non-surgical endodontic treatment is as high as 76% [8]. MSEO is in fact a specialized form of odontogenic sinusitis, caused by the spread of microbiota from the endodontic space of maxillary premolars and molars into the maxillary sinus; thus it should be considered as endodontic disease. If so, the objectives for MSEO treatment are the same as those for endodontic lesions management, and these are as follows: removal of microbiota, their by-products and infected debris from the root canal system, and prevention of reinfection [5]. Therefore the protocol of RCT is also crucial for effective treatment of MSEO disease.

Modern endodontic science has progressively emphasized and prioritized irrigation of the root canal system as a crucial part of every non-surgical RCT [9]. It has been proved that endodontic space cannot be effectively cleaned with only mechanical preparation [10]; therefore constant development of best root canal irrigation protocols and methods is one of the most important fields of scientific research in endodontics [9]. The application of ultrasounds (US) in root canal treatment was proven to be advantageous, as it enhances antimicrobial properties of sodium hypochlorite (NaOCl), which results in a higher rate of AP lesions healing [11]. Moreover, the passive ultrasonic irrigation (PUI) method is the most commonly used irrigation activation technique (IAT) within global specialist endodontic practice [12]. If PUI enhances AP healing and the microbiota isolated from dental and sinonasal sites are comparable [13], therefore it should be hypothesized that application of PUI in root canal irrigation protocol may be beneficial in MSEO treatment through non-surgical RCT. However, this correlation was not inspected up to this date.

The aim of the study was to inspect the influence of PUI on the healing of MSEO, when maxillary sinusitis was treated with non-surgical RCT.

## 2. Materials and Methods

**Figure 1:**
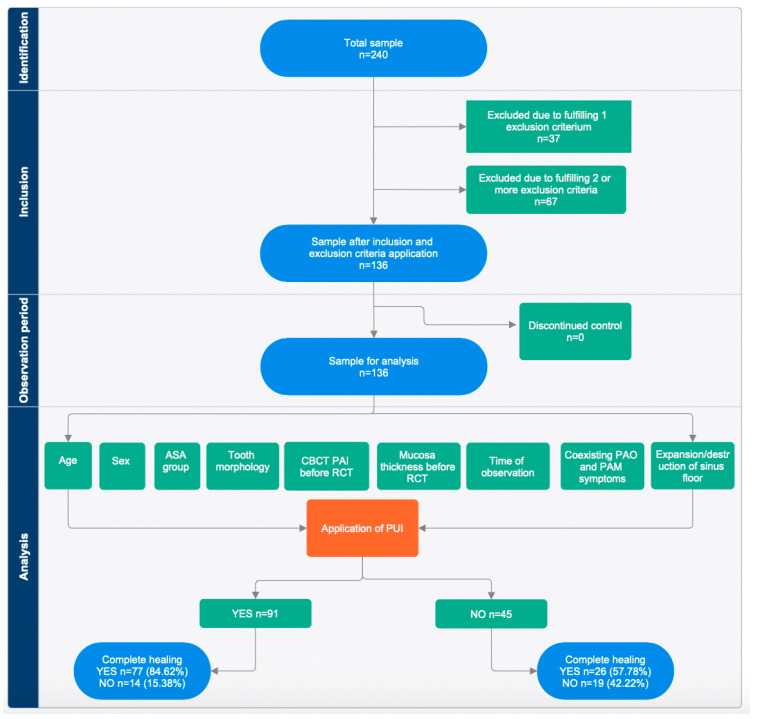
STROBE flow chart for retrospective observational study.

The study was designed as single-center, retrospective cohort study analyzing intraoperative data (application of PUI in root canal irrigation protocol) and the post-operative outcome of non-surgical endodontic treatment (CBCT pre- and post-op scans). All treatments were performed by the same operator—a specialist in endodontics (BC)—between 2016 and 2025. A total number of 240 medical records of 240 patients were analyzed. One hundred and thirty-six teeth entered the study due to inclusion and exclusion criteria. STROBE guidelines for reporting observational studies were applied (Figure 1).

### 2.1. MSEO Diagnostic Criteria

MSEO was diagnosed as the occurrence of PAO or/and PAM symptoms in the region of maxillary sinus adjacent to the tooth that underwent RCT with a coexisting AP lesion in the tooth apical area. PAM was diagnosed when the thickness of antral mucosa in preoperative CBCT scans was greater than 2 mm.

### 2.2. Inclusion Criteria (All of Following)

- Adult patients (Age > 18);
- ASA score 1 or 2 (no systemic diseases or mild systemic disease);
- MSEO: PAO and/or PAM signs in CBCT scans before the treatment, associated with tooth that underwent RCT;
- RCT performed by the same operator (BC) with the same treatment protocol;
- Asymptomatic or symptomatic cases with either dental or sinonasal symptoms.

### 2.3. Exclusion Criteria (Any of Following)

- Non-adult patients (Age < 18);
- ASA score 3 or higher;
- Pregnancy or nursing;
- Systemic antibiotic application during the course of endodontic treatment or/and in the period of observation;
- Bone remodeling medication applied during RCT or/and during period of observation;
- Any type of endodontic surgical treatment (e.g., resection) in the period of observation;
- Extraction of treated tooth;
- Laryngological non-surgical treatment 3 months prior to endodontic or/and during the course of RCT or/and in the period of observation (e.g., antibiotic);
- Laryngological/maxillofacial surgical treatment 1 year prior to endodontic treatment or/and in the period of observation;
- Reported trauma of treated tooth.

### 2.4. Medical Records and CBCT Scans Assessment Protocol

Medical records and CBCT scans were analyzed by two independent observers—a specialist in endodontics (BC) and a specialist in maxillofacial surgery (PS). Observers were calibrated using an image set of 4 randomly chosen CBCT scans. CBCT PAI index values, based on the linear measurements of AP diameter in CBCT scans, were noted for each observer (BC and PS) once per week for 5 weeks in order to assess inter-observer reproducibility. Additionally, intra-observer reproducibility was calculated for all observers with the same set of CBCT scans and the same time interval (set of 4 CBCT scans, assessed once in a week for 5 weeks). Intraclass correlation coefficient (ICC) type 2 by the Shrout and Fleiss model was used [14]. Medical records of the patients were screened for 6 factors: application of PUI in irrigation protocol during RCT, age, sex, general health condition (assessed with ASA physical status classification system), observation period, type of treated tooth. Pre-operative CBCT scans were analyzed to assess the size of the AP lesion scaled with CBCT-PAI index by Estrela et al. [15] and thickness of maxillary sinus mucosa in millimeters. The size of AP lesion was measured as the biggest diameter of periapical radiolucency in axial, coronal, or sagittal cross-sections. The thickness of proliferated maxillary sinus mucosa was measured at the greatest measurement in axial, coronal, or sagittal cross-sections. Then, CBCT control scans were assessed for the same factors. The observers were blinded to treatment allocation, follow-up status, and factors studied. The examinations were performed using Carestream CS 9300 Cone Beam 3D System (Carestream Health Inc., Rochester, New York, NY, USA) with image acquisition parameters: 60–90 kV, 2–15 mA and voxel size 90–500 μm. Small or medium FOV (5 cm × 5 cm to 17 cm × 13.5 cm) were used, and control CBCT FOVs matched those from pre-op CBCT scans. CBCT scans were assessed in a dark room on Baecon 24″ HL2416SH medical monitor (Shenzhen Beacon Display Technology Co., Ltd., Shenzhen, China) with CS 3D Imaging Software version 8.0.31 (Carestream Health Inc., Rochester, New York, NY, USA). Complete healing was defined as total resolution of AP lesion (CBCT PAI = 0), coexisting with complete healing of PAO and PAM symptoms (thickness of maxillary sinus mucosa between 0 and 2 mm) and resolution of all clinical symptoms.

### 2.5. Endodontic Treatment Protocol

All treatments were performed by the same operator, using the same equipment in the same clinic site. All patients signed informed consent. Patient was anesthetized with infiltration anesthesia using 4% articaine with 1:200,000 adrenaline (Septanest 1:200, Septodont, Saint-Maur-des-Fossés, France). In every treatment the tooth was isolated with a rubber dam (Hygenic Dental Dam Kit, Coltene/Whaledent, Altstätten, Switzerland), and a dental operating microscope (DOM) was used (Leica M320, Leica Microsystems, Wetzlar, Germany). Glide-path preparation was performed with ISO 08 and 10 C-pilots (VDW, Frankfurt am Main, Germany). All canals were initially hand prepared with H-files (VDW, Munich, Germany) with a step-back technique to a size 20.02, followed by a crown-down technique using Endostar E3 rotary instruments (PolDent, Warsaw, Poland) up to 30.04 size and MTwo 35.04 rotary file, 40.04, or 45.04 (depending on the size of IAF) (VDW, Munich, Germany). Minimum size of MAF was 35.04. Working length (WL) was assessed with Raypex 6 apex locator (VDW, Munich, Germany). Every canal was copiously irrigated with 5.25% sodium hypochlorite (NaOCl) (Chloraxid, Cerkamed, Stalowa Wola, Poland) during mechanical preparation with hand and rotary files. Final irrigation protocol was as follows: 5.25% NaOCl 5 mL/canal, 40% citric acid (CA) 5 mL/canal, and final flush with 2% chlorhexidine (CHX) 5 mL/canal. In part of the cases, the PUI method was applied, with activation of NaOCl during mechanical preparation and NaOCl and CA in final irrigation protocol. The allocation of cases to PUI and non-PUI groups was equipment-dependent. All treatments done before 2019 were performed without PUI, and the reason was that this equipment was not available in the practice at that time. After 2019 PUI was introduced as the daily clinical routine and applied in most of the cases; however in some of the treatments conducted after 2019 PUI was not applied due to ultrasonic unit failure, lack of endo-chucks or ultrasonic files. PUI was performed with K-file size 30.02 (Woodpecker Dental, Guilin, Guangxi, China), mounted on 120° E1 endo-chuck (Woodpecker Dental, Guilin, China) using Woodpecker UDS-A LED ultrasonic unit (Woodpecker Dental, Guilin, China). Canals were dried with sterile paper points (MetaBiomed, Cheongju-si, Chungcheongbuk-do, Republic of Korea) and obturated with a vertical compaction of warm gutta-percha (GP) with the continuous wave of condensation technique, using master apical gutta-percha cones (MetaBiomed, Cheongju-si, Chungcheongbuk-do, Republic of Korea) with AHplus sealer (Dentsply Sirona, Charlotte, NC, USA). Super Endo B&L system was used for the obturation (B&L Biotech, Fairfax, VA, USA). Intraoral radiograph (RVG) was taken. Patient was notified to return to control CBCT after 6, 12, and 24 months.

## 3. Results

### 3.1. Study Group Characteristics

Mean age of patients included in the study was 44.75 (±13.46) years, with 47.06% female (*n* = 64) and 52.94% male patients (*n* = 72). Most of the patients were classified as ASA I group (83.82%, *n* = 114). Complete healing was observed in 75.74% of the cases (*n* = 103). The tooth most frequently associated with MSEO was the first upper molar (52.21%, *n* = 71). In 92.65% of the cases (*n* = 126), PAO and PAM symptoms coexisted. Destruction and expansion of maxillary sinus floor was observed in 40.44% of cases (*n* = 55). Most of the control CBCTs were made between 13 and 24 months after the treatment (52.21%, *n* = 71). PUI was applied in most of the treatments (66.91%, *n* = 91). Total characteristics of the study group are presented in Table 1.

### 3.2. The Influence of PUI on MSEO Healing

**Figure 2:**
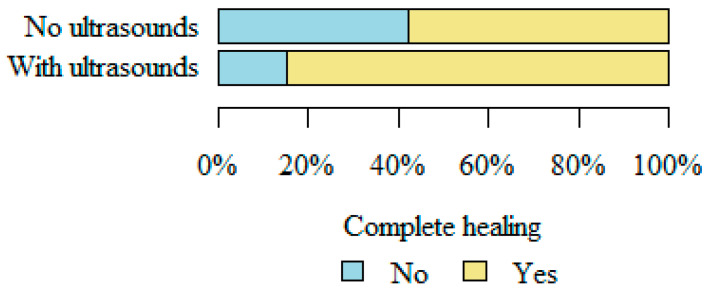
The influence of ultrasonic application on the healing of MSEO.

Application of PUI in the irrigation protocol correlated with higher rates of the MSEO healing (*p* = 0.001; 95% CI [1.768; 9.136]). When PUI was introduced in RCT, complete healing of MSEO was observed in 84.62% of the cases in comparison to 57.78% healing rate, when ultrasonic activation was not performed (Table 2) (Figure 2). Univariate logistic regression models showed that PUI was independently associated with higher odds of radiological resolution of MSEO (OR = 4.019). The same trend was noted when multiple logistic regression models were applied (OR = 12.388) (Table 3).

Mean, standard deviation, median, quartiles, and range of quantitative variables were shown.

For qualitative variables, absolute and relative frequencies (*N* and %) were reported.

Chi-squared test (with Yates correction for 2 × 2 tables) or Fisher’s exact test (in case of low expected values) was used for comparisons of qualitative variables between groups.

Univariate and multiple logistic regression was employed to model the potential impact of predictors on a dichotomous variable. ORs (odds ratios), alongside the 95% confidence intervals, were presented. The significance level was set to 0.05.

Generalized variance inflation factor (GVIF) was reported to ensure model robustness (Table 4).

### 3.3. Statistical Analysis

The inter-rater reliability of the observers, calculated with ICC2, was 0.826; intra-rater reliability calculated with ICC2 for observer 1 (BC) was 0.868 and for observer 2 (PS) was 0.844, which showed good agreement.

All the analyses were conducted in R software, version 4.5.2 [16].

## 4. Discussion

PUI is most widely used IAT among endodontists worldwide [12]. The method is defined as intermittent, 10–30 s US activation combined with constant donation of fresh irrigant [17]. Freely oscillating US file causes a unique phenomenon called acoustic streaming [18], which results in better distribution of the irrigants into all elements of endodontic space, creating “tsunami irrigation” [19]. Part of the ultrasonic energy is also converted to heat [20], which results in the rise of temperature of the solution, increasing the action of NaOCl [21]. Although it was proved that application of PUI enhances healing of AP after non-surgical RCT [11], the potential influence of this agitation method on MSEO healing was not inspected up to this date. We designed the research as a single-center study with only one endodontist performing all the treatments, along with the use of strict exclusion criteria aimed at reducing operator bias and variables, which could have influenced the results. Previous studies on MSEO healing not only included a relatively small number of treated cases, but also those treatments were performed by multiple operators with different clinical experience and skills [22]. This was implicated in a rather low rate of treatment success (30–60%) [22]. Moreover, our clinical and radiological healing criteria were stricter, as only coexistence of total resolution of AP lesion and thickness of mucosa 0–2 mm were classified as “complete healing”. With that defined, we confirmed that the rate of healing of MSEO after non-surgical RCT is high (75.74%), which was consistent with the results from our previous studies. It should be emphasized that the rate of MSEO healing was relatively high, even when PUI was not applied in irrigation protocol (57.78%). Therefore this method should be described as the one that enhances the healing of MSEO and by this, PUI should be considered as a potentially beneficial, additional factor that improves clinical performance. The specificity of the retrospective design of the study limited its randomization; moreover, we used convenience sampling, which can introduce selection bias and limit the ability to generalize findings to the broader population. The current research should be treated as a pilot study for conducting a randomized controlled trial on this subject in the future, involving ENT examination prior to endodontic treatment.

## 5. Conclusions

Within the limitations of this retrospective study, PUI application in irrigation protocol was associated with higher rates of MSEO healing (*p* = 0.001; 95% CI [1.768; 9.136]). Once again we have proved that the rate of MSEO healing after non-surgical RCT is high (75.74%). When PUI was applied in RCT, complete healing of MSEO was observed in 84.62% of the cases, and this method of agitation was independently associated with higher odds of radiological resolution of MSEO in both univariate and multiple logistic regression models (OR = 4.019 and OR = 12.388, respectively).

## Figures and Tables

**Table 1:** Study group characteristics.

Parameter		Total (*N* = 136)
Age [years]	Mean (SD)	44.75 (13.46)
	Median (quartiles)	42 (35–52)
	Range	20–74
	*n*	136
Sex	Female	64 (47.06%)
	Male	72 (52.94%)
ASA	ASA I	114 (83.82%)
	ASA II	22 (16.18%)
Complete healing	No	33 (24.26%)
	Yes	103 (75.74%)
Application of US in irrigation protocol	No ultrasounds	45 (33.09%)
	With ultrasounds	91 (66.91%)
Tooth morphology	First premolar	9 (6.62%)
	Second premolar	19 (13.97%)
	First molar	71 (52.21%)
	Second molar	36 (26.47%)
	Third molar	1 (0.74%)
CBCT PAI before treatment	0	7 (5.15%)
	1	1 (0.74%)
	2	5 (3.68%)
	3	27 (19.85%)
	4	56 (41.18%)
	5	40 (29.41%)
Thickness of mucosa before the treatment	Up to 5 mm	44 (32.35%)
	5–10 mm	44 (32.35%)
	Over 10 mm	48 (35.29%)
Time of observation	6–12 months	48 (35.29%)
	13–24 months	71 (52.21%)
	Over 24 months	17 (12.50%)
Coexisting of PAO and PAM symptoms	No	10 (7.35%)
	Yes	126 (92.65%)
Expansion/destruction of sinus floor bone	No	65 (47.79%)
	Expansion	16 (11.76%)
	Expansion + destruction	55 (40.44%)

**Table 2:** Complete healing of MSEO with and without the application of PUI method.

Complete Healing	Application of US in Irrigation Protocol		*p*
	No Ultrasounds (*N* = 45)	With Ultrasounds (*N* = 91)	
No	19 (42.22%)	14 (15.38%)	*p* = 0.001
Yes	26 (57.78%)	77 (84.62%)	

**Table 3:** The influence of PUI application on MSEO healing in univariate and multiple models.

Trait		*N*	*n*	Univariate Models				Multiple Model			
				Parameter	95%CI		*p*	Parameter	95%CI		*p*
Application of PUI in irrigation protocol	No PUI	45	26	1	ref.			1	ref.		
	With PUI	91	77	4.019	1.768	9.136	0.001 *	12.388	1.373	111.782	0.025 *
Age [years]		-	-	0.956	0.928	0.986	0.004 *	0.985	0.912	1.064	0.704
Sex	Female	64	53	1	ref.						
	Male	72	50	0.472	0.208	1.071	0.073				
General health condition	ASA I	114	87	1	ref.						
	ASA II	22	16	0.828	0.295	2.325	0.72				
Tooth morphology	Tooth 6	71	48	1	ref.						
	Tooth 4	9	9	---	---	---	---				
	Tooth 5	19	17	4.073	0.867	19.135	0.075				
	Tooth 7 or 8	37	29	1.737	0.687	4.39	0.243				
CBCT PAI before the treatment	0	7	4	1	ref.						
	1–2	6	3	0.75	0.084	6.71	0.797				
	3	27	22	3.3	0.554	19.653	0.19				
	4	56	40	1.875	0.377	9.336	0.443				
	5	40	34	4.25	0.753	23.981	0.101				
Thickness of maxillary sinus mucosa before the treatment	Up to 5 mm	44	40	1	ref.			1	ref.		
	5–10 mm	44	30	0.214	0.064	0.717	0.012 *	0.539	0.026	11.008	0.688
	Over 10 mm	48	33	0.22	0.067	0.727	0.013 *	0.611	0.042	8.924	0.719
Time of observation	6–12 months	48	44	1	ref.			1	ref.		
	13–24 months	71	48	0.19	0.061	0.592	0.004 *	3.855	0.261	56.922	0.326
	Over 24 months	17	11	0.167	0.04	0.695	0.014 *	7.942	0.355	177.651	0.191
Coexisting of periapical lesion and thickness of mucosa	No	10	7	1	ref.						
	Yes	126	96	1.371	0.334	5.636	0.661				
Expansion/destruction of sinus floor bone	No	65	48	1	ref.						
	Expansion	16	13	1.535	0.389	6.051	0.541				
	Expansion + destruction	55	42	1.144	0.498	2.63	0.751				

*N*—group size, *n*—cases of complete healing; * statistically significant (*p* < 0.05). Only variables that were statistically significant in the univariate analysis were included in the multivariate analysis.

**Table 4:** Generalized variance inflation factor (GVIF) calculated for the variables.

Trait	GVIF *
Application of US in irrigation protocol	1.16
Post-op thickness of mucosa [mm]	1.20
Age [years]	1.21
Time of observation	1.38
Pre-op thickness of mucosa	1.10

* GVIF < 5 indicates no overfitting.

## Data Availability

The raw data supporting the conclusions of this article will be made available by the authors on request.

## Abbreviations

The following abbreviations are used in this manuscript:

AP	Apical Periodontitis
MSOO	Maxillary Sinusitis of Odontogenic Origin
AAE	American Association of Endodontists
MSEO	Maxillary Sinusitis of Endodontic Origin
RCT	Root Canal Treatment/Therapy
US	Ultrasounds
NaOCl	Sodium hypochlorite
PUI	Passive Ultrasonic Irrigation
IAT	Irrigation Activation Techniques
CBCT	Cone Beam Computed Tomography
PAO	Periapical Periosteitis
PAM	Periapical Mucositis
ASA	American Society of Anesthesiologists
ICC	Intraclass correlation coefficient
DOM	Dental Operating Microscope
IAF	Initial Apical File
MAF	Master Apical File
WL	Working Length
CA	Citric Acid
CHX	Chlorhexidine
RVG	Radiovisiography
OR	Odds Ratio
ENT	Ear Nose and Throat
